# Well-Being at Work: Burnout and Engagement Profiles of University Workers

**DOI:** 10.3390/ijerph192315436

**Published:** 2022-11-22

**Authors:** Pablo González-Rico, Eloísa Guerrero-Barona, Maria José Chambel, Mónica Guerrero-Molina

**Affiliations:** 1 Business Management Department, University CEU San Pablo, 28003 Madrid, Spain; 2 Department of Psychology, University of Extremadura, 06006 Badajoz, Spain; 3 Facultade de Psicologia, Universidade de Lisboa, 1649-013 Lisbon, Portugal

**Keywords:** well-being, engagement, burnout, happiness, satisfaction with life

## Abstract

The main objective of this study is to analyze well-being at work, considering burnout and engagement distributed in profiles, and to observe how they relate to well-being outside work. The data came from a representative sample of workers (*n* = 565) at the University of Extremadura (Spain), both teaching and research academic staff (TRAS) and service and administrative staff (SAS). We performed the data analysis by using latent profile analysis, and the results show evidence that workers from both groups were distributed across four profiles. As expected, we verified that workers in the profile with high burnout and low engagement had lower well-being outside work than workers in the profile with high engagement and low burnout. We also observed that engagement mitigated the negative effects of burnout on workers in profiles with moderate levels of burnout, who showed better well-being outside work when they had higher engagement. These differences are discussed, and their practical implications and suggestions for future research are provided.

## 1. Introduction

It is undeniable that society is constantly changing due to the need to adapt to the environment and, exacerbated by the period of global economic and sanitary crisis in which we find ourselves, we are currently undergoing profound changes in all areas of work, including universities [1,2]. This is one of the reasons why a considerable increase in professionals’ perception of chronic stress in their jobs is now manifesting itself [3,4]. Today, this phenomenon is considered to be the most important psychosocial harm in society and has been called burnout syndrome [5]. However, it was in the late 1990s when, with the emergence of positive psychology, the research in this field changed direction. The search for preventive factors began to gain importance in research in favour of harmful factors, giving rise to the concept of engagement, which was finally considered to be the antithesis of burnout [5,6]. Burnout is a three-dimensional syndrome characterized by emotional exhaustion, depersonalization and professional efficacy [7], while engagement is “a positive psychological state related to work that is characterized by vigor, dedication and absorption or concentration at work” [8] (p. 79). There are a lot of studies that have addressed the relationship between burnout and engagement, showing the significantly negative relationship between them [9,10,11,12].

Therefore, the first objective of this work was to test the simultaneous occurrence of burnout and engagement, that is, to verify that there are two related but independent constructs that can co-occur in the same employee [10,13,14]. Previous studies have identified different profiles where both burnout and work engagement occurred simultaneously in students of high schools, highly educated employees or workforces [15,16,17]. However, an innovative contribution of this study is that a sample of university workers was used: both teaching and research academic staff (TRAS) and service and administrative staff (SAS).

We studied these two populations with distinct functions and tasks working in the same context, i.e., a university. Previously, studies developed in a university context have analyzed privileged students, while TRAS is a little-studied educational group as far as labor welfare is concerned, while SAS is practically unexplored. A distinction is made between the two samples because, despite working in the same university, they have different demands, functions and even motivations. Changes in education laws have led to a significant increase in the functions of TRAS, so the need to be more competitive with fewer resources is perceived as stressful [18]. SAS is a group with an occupation characterized by routine, simple and repetitive jobs [19], causing high levels of stress [20,21,22]. These stress situations negatively interfere in workers’ well-being [23]. Therefore, as the current health crisis and the upcoming economic crisis are having a negative impact on the mental health of the population, and the importance of having a job at the current moment is vital, we intend to provide a positive view of the situation, analyzing the importance of engagement at work, and the relationship of work aspects to wellbeing outside the work context, trying to provide a starting point on how to approach our view of work.

### 1.1. Well-Being at Work: Burnout and Engagement

Maslach et al. considered burnout to be a syndrome characterized by emotional exhaustion, depersonalization, and reduced professional accomplishment [24]. Emotional exhaustion relates to emotional resource loss due to user demands. Depersonalization refers to negative and cynical attitudes toward users. Finally, the lack of professional fulfillment refers to the tendency to evaluate one’s own work from a negative standpoint [25]. Although studies into burnout began in human service professions [26,27,28], nowadays, it has spread to virtually all professional groups, such as the military [29,30], prison staff [31,32] and non-university teachers [33,34,35]. However, the university education sector has been little studied so far in relation to teaching and research academic staff (TRAS), though evidence has been found that, as in other educational levels, burnout indicators are indeed present [22,36], while, with respect to service and administrative staff (SAS), there are still too few studies examining burnout, although those that have been carried out show that this population also presents with the syndrome [37,38].

Engagement is a concept that grew out of the need to find a concept in contrast to that of “burnout”, but it is not exactly the opposite of burnout because the two concepts represent different dimensions of well-being at work, are evaluated differently and may even co-occur simultaneously [10,14]. In fact, burnout and engagement are two different dimensions of well-being at work and can be developed simultaneously in the same situation [10,13]. The definition is “a psychological state work-related, positive and characterized by vigor, dedication and absorption or concentration at work” [8] (p. 79). Vigor is characterized by high levels of energy, stamina and mental activation while working, keeping the belief in what you do and overcoming any present obstacles. Dedication refers to the implication that the person has in their work, to a high pride in the work they do and engagement in work tasks. Finally, absorption refers to concentration at work, and the enjoyment in performing the work tasks, to the point that it can be difficult to disconnect from the work itself [13].

We assumed that burnout and engagement could occur simultaneously as indicators of well-being at work, inspired by previous studies [10,13], showing that they are two independent constructs that can co-occur in the same employee. On the other hand, the role of profile research is to identify groups of workers to understand how they behave in their organization. For example, although no previous studies have been found that analyzed worker profiles in the university working population, four different profiles were encountered by Tuominen-Soini and Salmela-Aro [17] in a sample of high school students: students with high engagement and low burnout (engaged profile), students with high engagement and high exhaustion (engaged–exhausted profile), students characterized by pronounced cynicism only (cynical profile) and students with low engagement and high burnout (burned-out profile). Students in the engaged and engaged–exhausted profiles were performing well in school, although the “engaged-exhausted” profile were more stressed and preoccupied with possible failures. Students in the last two profiles had lower academic achievement and less value to the school, although the “burned-out” students were more stressed, exhausted and depressed. Therefore, in this study we hope to identify four types of worker profiles in which burnout and engagement can occur simultaneously, looking at all four possible options.

**H1:**  *Four types of well-being profiles, with the following burnout and engagement, will emerge in the two populations studied (TRAS and SAS): (1) a profile with high engagement and low burnout scores; (2) a profile with high burnout and low engagement scores; (3) a profile with low burnout and engagement scores; and (4) a profile with high burnout and engagement scores.*

### 1.2. Well-Being at Work–“Context-Free” Well-Being

The literature distinguishes between well-being at work and employee well-being outside work, namely satisfaction about your life in general [39]. In Spain, the Law on Occupational Health and Safety [40] states that burnout should be studied with the aim of improving health and quality of life. The demands of work can affect not only efficiency at work, but also the employees’ quality of life outside work [41].

Work is one of the most important factors in the lives of many people [42], so it has been shown that well-being at work spills over to overall well-being, i.e., burnout and engagement have influence on “context-free well-being” [43]. According to conservation of resources theory [44], burnout results from the loss of energetic resources after heavily investing in work without appropriate gains in return. Moreover, the conservation of resources (COR) theory maintains that initial resource loss is likely to lead to loss spirals, i.e., to future losses of other resources, and to subsequent deteriorated well-being [45]. Indeed, research shows that burnout is related to several resource losses, which undermines the individual’s coping capabilities and means that this negative work-related state will spill over and generalize into negative context-free states. On the other hand, COR theory also assumes the possibility of gain spirals, i.e., those who possess resources are likely to gain more resources over time [46]. Being engaged at work may further increase personal resources and may thereby also spill over to context-free well-being.

We use the term “context-free well-being” to refer to indicators of overall well-being outside work [47]. The “context-free well-being” can be measured by satisfaction with life, positive and negative affect, depression, happiness, anxiety and self-esteem [48]. In the present study, we used satisfaction with life and happiness to measure “context-free well-being”. According to the concept of happiness, happy people achieve success in multiple domains of life, including health and work [49,50]. Those who perceive themselves as happy people tend to face life in a positive and adaptive way, with a firm attitude to face any eventuality that may occur [51]. With respect to satisfaction with life, this has been considered one of the components of subjective well-being, as it reflects the degree to which a person evaluates their own life in a favorable way [52,53].

Studies have shown that burnout is negatively related to happiness [54,55] and satisfaction with life [56,57]. Furthermore, other studies have shown that engagement is significantly and positively related to happiness [58,59] and satisfaction with life [60,61]. In short, a low level of well-being at work causes negative collateral effects on the individual’s life in general outside of work, leading to low satisfaction with life [43] and low happiness [62]. There are studies that have analyzed the relationship between different profiles with respect to well-being at work [63,64]; however, no studies that analyze the relationship between the different profiles of burnout and engagement with respect to “context-free well-being” were found. Therefore, in order to make a contribution to the literature and taking as inspiration the conservation of resources theory previously discussed [44], it may be that those workers with a profile of high scores on engagement and low scores on burnout have the highest “context-free well-being”; conversely, workers with a profile of low scores on engagement and high scores on burnout may have the lowest well-being outside work. Finally, workers with a profile of high scores on engagement and high scores on burnout could have more “context-free well-being” than those with a profile of low scores on engagement work and low scores on burnout, since the engagement could act as a buffer against burnout and continue the gains spiral proposed by Hobfoll [44].

**H2:**  *The profile with high levels of engagement and low levels of burnout will have higher levels of happiness and life satisfaction than the profile with low levels of engagement and high levels of burnout in the two populations studied (TRAS and SAS).*

**H3:**  *The profile with high levels of engagement and high levels of burnout will have higher levels of happiness and satisfaction with life than the profile with low levels of engagement and low levels of burnout in the two populations studied (TRAS and SAS).*

## 2. Materials and Methods

### 2.1. Participants and Procedure

The total sample consisted of 585 workers at the University of Extremadura (see Table 1): 266 service and administrative staff (45.47%) and 319 teaching and research academic staff (54.53%), referred to from now on as SAS and TRAS, respectively. This was a representative sample of all the workers at the University of Extremadura selected by simple random sampling, and all of them completed the questionnaires voluntarily without receiving any type of incentive. In terms of gender, a good balance was shown (52.8% of men compared to 47.2% of female participants). In reference to the age of the participants, most were between 41–50 years old (215 = 36.8%). With respect to the years of tenure, most had been working for over 20 years (178 = 30.4%) or 5 to 15 years (176 = 30.1%). The sample size was determined to estimate a variable ratio with a precision of 0.05 and a confidence of 95%, considering the hypothesis of maximum variance.

Once the sample was made, and with an institutional and ethical agreement with the university achieved, we sent the assessment instrument using the instrument “formulary” of Google Drive, along with the presentation of the research objectives. Participants responded to an online questionnaire voluntarily which guaranteed anonymity. After four months of the initial release of the questionnaires, the total sample required was.

### 2.2. Instruments

Burnout. This was measured with the Maslach Burnout Inventory G-S, developed by Schaufeli, Leiter, Maslach and Jackson in [65], which is made up of 22 items. It consists of three dimensions that make up the syndrome: emotional exhaustion, cynicism and professional efficacy. The items are related to the feelings of people at work over the previous year. The response scale is Likert 0 “never” to 6 “every day”. The internal consistency of Cronbach’s alpha reached by each subscale was: emotional exhaustion, α = 0.90 for SAS and α = 0.88 for TRAS; cynicism, α = 0.75 for SAS and α = 0.74 for TRAS; professional efficacy, α = 0.79 for SAS and α = 0.82 for TRAS.

Engagement. This was measured with the 9-item short version of the Utrecht Work Engagement Scale (UWES-9), developed by Schaufeli, Bakker and Salanova in [66]. The three dimensions that make up engagement—namely, vigor, dedication and absorption—were considered. The items relate to the feelings of the people in their work over the previous year. The response scale is Likert 0 “never” to 6 “every day”. The internal consistency of Cronbach’s alpha reached by each subscale was: vigor, α = 0.84 for SAS and α = 0.86 for TRAS; dedication, α = 0.91 for SAS and α = 0.88 for TRAS; and absorption, α = 0.81 for SAS and α = 0.79 for TRAS.

Happiness. This was measured with the Subjective Happiness Scale, developed by Lyubomirsky and Lepper in [67]. It consists of 4 items, all related to people’s feelings of happiness in their daily lives. The response scale Likert is 1 to 7, but the meanings of the values of the scale vary for each item (Item 1: 1 = not a very happy person, 7 = a very happy person; item 2: 1 = less happy, 7 = more happy; item 3: 1 = not at all, 7 = a great deal; item 4: 1 = not at all, 7 = a great deal). The internal consistency of Cronbach’s alpha reached by the scale was satisfactory (α = 0.84 for SAS and α = 0.77 for TRAS).

Satisfaction with life. This was measured using the Scale of Satisfaction with Life developed by Diener, Emmons, Laren and Griffin [68], which has shown good psychometric properties in previous studies in the Spanish language [69]. It consists of 5 items related to the degree of satisfaction of people with their lives. The response scale is Likert 1 “completely disagree” to 7 “completely agree”. The internal consistency of Cronbach’s alpha reached by the scale was α = 0.94 for SAS and α = 0.88 for TRAS.

### 2.3. Control Variables

Finally, after reviewing the scientific literature, it was considered appropriate to control sex, age and tenure, since several studies showed that these factors could have some influence on workers’ well-being [70,71]. For the present study, these variables were measured through a specifically designed, self-reported questionnaire.

### 2.4. Procedure of Data Analyses

The data analysis of this research was conducted in four phases. In the first, we made a confirmatory factor analysis to assess the discriminant validity of the four scales used, and to test the common variance of the results [72]. Specifically, we compared how well a 4-factor model fit a single factor model, in which all items were grouped into a single latent variable. In the second, we performed a Latent Profile Analysis (LPA) using the maximum likelihood method through the MPlus 7.11 program [73] to identify the profiles of the relationship between burnout and engagement. The LPA categorizes the latent variables to identify groups of individuals with similar relationship patterns between variables. According to Nylund, Asparouhov and Muthén [74], this analysis is performed through an interactive process in which, firstly, a model is estimated from two profiles and then more profiles are successively added, up to the optimal number. Each model is evaluated using criteria designated for this: (a) Bayesian sample-adjusted information criterion [75], (b) the plausibility bootstrap [76], (c) the number of subjects in each profile, and (d) the probabilities of belonging to each profile and not another [77]. The SABIC is used to select the best fitting model and the least number of parameters from a set of non-hierarchical models. Nylund et al. [74] suggest that the best fit to the data is acquired when (a) show a lower SABIC, (b) are profiles with a reduced number of subjects, and (c) are clearly defined profiles, i.e., when subjects have a high probability of belonging to the profile in which they were placed and a low probability of belonging to another profile. In the third phase, we conducted an analysis of variance (ANOVA) to assess the uniqueness of the profiles generated by the LPA. We placed profiles as an independent variable, and burnout and engagement as dependent variables. To allocate a specific denomination to each of the profiles, a comparison was made of the average values found for burnout and engagement in each profile. Finally, the fourth stage of the procedure was carried out to test hypothesis 2, i.e., whether each of the profiles found differed with respect to the levels of welfare outside work (happiness and satisfaction with life). For this, we conducted the ANCOVA analysis, with gender, age and years of work experience as the control variables.

## 3. Results

### 3.1. Confirmatory Factor Analysis

Through confirmatory factor analysis, we verified that our four-factor model showed an adequate fit to the data, analyzed separately in both samples [*x*^2^ (282) = 732.93, *p* > 0.001, SRMR = 0.07, CFI = 0.93, TLI = 0.92, RMSEA = 0.07 for SAS; and *x*^2^ (282) = 756.43, *p* > 0.001, SRMR = 0.07, CFI = 0.92, TLI = 0.91, RMSEA = 0.07 for TRAS], as compared to the one-factor model, in which all items were grouped into a single latent variable [*x*^2^ (292) = 2507.64, *p* > 0.001, SRMR = 0.10, CFI = 0.64; TLI = 0.60, RMSEA = 0.17 for SAS; and *x*^2^ (292) = 2243.79, *p* > 0.001, SRMR = 0.10, CFI = 0.66, TLI = 0.62, RMSEA = 0.15 for TRAS]. We thus conclude that our theoretical model provided a best fit to the data (Δ*x*^2^ (10) = 1774.71, *p* < 0.001 for SAS; and Δ*x*^2^ (10) = 1487.36, *p* < 0.001 for TRAS).

The means, standard deviations and correlations between variables revealed that the correlation coefficients between burnout (exhaustion, cynicism and professional achievement), engagement (vigor, dedication and absorption), and happiness and life satisfaction were significant in both samples (see Table 2). Almost all variables were significantly related to each other in both professional groups, except for the relations of “cynicism” with “absorption” and “happiness”, since this was not a significant relationship in TRAS, while in the SAS a significant relationship occurred.

### 3.2. Latent Profile Analyses

**Figure 1:**
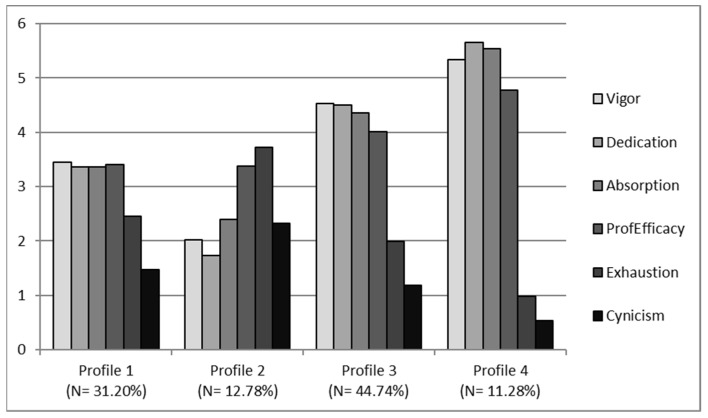
Profile distribution in SAS.

**Figure 2:**
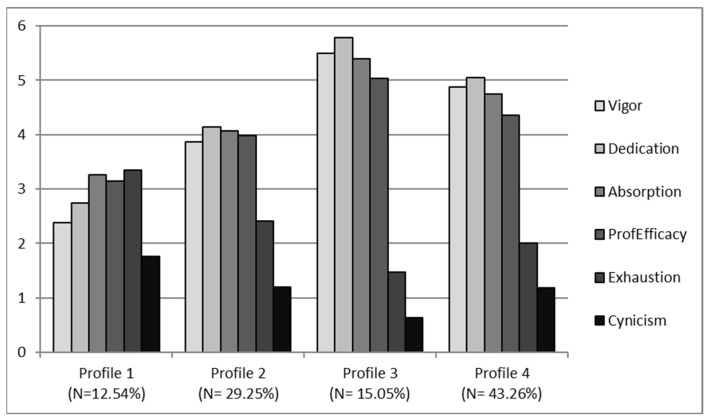
Profile distribution in TRAS.

We began the latent profile analysis by specifying a two-profile model and adding more profiles until no convergence problems were found [75]. The ideal model includes four profiles for each of the samples of the study and the rationale for the choice of the final model is presented in Table 3, Table 4 and Table 5. We first checked that this was the model that had the lowest SABIC (3690.74 for SAS, and 4456.50 for the TRAS) (see Table 2). The results in Table 3 indicate that the number of cases in each profile was sufficient to ensure the subjects stayed in the profile (range = 30 to 119 in SAS, and 40 to 138 in TRAS). Membership probabilities associated with each profile indicate that the four-profile model differs from the others (see Table 4). Thus, the likelihood that individuals belong to designated profiles is very high (0.926–0.974 in SAS, and 0.899–0.969 in TRAS), as compared with the odds of individuals belonging to any other profile (0.00 to 0.054 in SAS and 0.00 to 0.085 in TRAS). Finally, Figure 1 and Figure 2 are built based on the averages of burnout and engagement.

The results of the ANOVAs, comparing the levels of the subscales of burnout and engagement between profiles, verified that there were significant differences between them in both samples. In the SAS: exhaustion (*F* (3, 44.35) = 68.77, *p* < 0.001); cynicism (*F* (3, 19.05) = 26.53, *p* < 0.001); professional efficacy (*F* (3, 17.61) = 54.37, *p* < 0.001); vigor (*F* (3, 81.66) = 293.08, *p* < 0.001); dedication (*F* (3, 106.74) = 430.07, *p* < 0.001); and absorption (*F* (3, 69.22) = 215.40, *p* < 0.001); and in the TRAS: exhaustion (*F* (3, 30.17) = 34.73, *p* < 0.001); cynicism (*F* (3, 9.20) = 10.40, *p* < 0.001); professional efficacy (*F* (3, 28.02) = 77.04, *p* < 0.001); vigor (*F* (3, 95.78) = 458.80, *p* < 0.001); dedication (*F* (3, 86.87) = 660.05, *p* < 0.001); and absorption (*F* (3, 43.95) = 92.13, *p* < 0.001). Profiles 1, 2, 3 and 4 differed quantitatively in terms of overall levels of burnout (exhaustion, cynicism and professional efficacy) and engagement (vigor, dedication and absorption). We relied on the results of the ANOVA and post hoc comparisons to name each of the profiles.

After seeing these results, it is clear that, for both samples, the profile distribution was very similar. In the SAS, engagement was significantly higher in profile 4 (11.28% of the sample) with respect to other profiles, and with lower levels of burnout. For this reason, it was decided to name profile 4 “Engaged”. Profile 3 included most of the sample (44.74%) and had a moderately high level of engagement and a moderately low level of burnout, so it was named “Moderate well-being”. Profile 1 (31.20% of the sample) had almost balanced moderate levels of engagement and burnout, so it was given the name “Balanced”. Finally, profile 2 (12.78% of the sample) had low levels of engagement and higher burnout, so this profile was named “Burned-out”. In the TRAS, engagement was significantly higher in profile 3 (15.05% of the sample), with very high levels of engagement and very low burnout, so it was given the name “Engaged”. Profile 4 included most of the sample (43.26%) and had moderately high levels of engagement and moderately low burnout, although not as low as in the previous profile, which is why it was decided to name profile 4 “Moderate well-being”. Profile 2 (29.25% of the sample) had balanced moderated levels of engagement and burnout, so it was called “Balanced”. Finally, profile 1 (12.54% of the sample) had lower levels of engagement and higher levels of burnout, so this profile was named “Burned-out”.

Hypothesis 1 postulates that there are four profile groups with different patterns of burnout and engagement. It must be concluded that Hypothesis 1 was partially supported. According to our assumption, there are four groups of profiles characterized by different levels of burnout and engagement in both samples. As expected, we observed a profile with high engagement and low burnout and another with high burnout and low engagement. However, the remaining two profiles are different from what was expected: in both profiles, engagement levels predominated over burnout levels. We did not find profiles with low levels of burnout and engagement, nor profiles with high levels of burnout and engagement.

### 3.3. Analysis of Covariance

The analysis of the results of the correlations found a justification for controlling gender, age and tenure in our hypotheses. To do this, we decided to test our hypothesis using the one-way ANCOVA test, with the profile belonging to each subject as the independent variable, and gender, age and years of practice as covariates. Earlier one-way ANCOVA tests were performed to check the underlying theory, including normality, the homogeneity of variance, and the homogeneity of regression diversions. These principles were met.

The one-way ANCOVA revealed significant differences between the profiles in: happiness, both in SAS (*F* (3, 19.60) = 17.92, *ETA* = 0.17, *p* < 0.001) and TRAS (*F* (3, 26.50) = 31.47, *ETA* = 0.23, *p* < 0.001); and satisfaction with life, both in SAS (*F* (3, 30.67) = 33.26, *ETA* = 0.28, *p* < 0.001) and TRAS (*F* (3, 37.78) = 75.70, *ETA* = 0.42, *p* < 0.001). Post hoc tests for comparison profiles were also conducted. The results of this post hoc analysis can be seen in Table 5 and Table 6. According to the second hypothesis, it was expected that profiles with high levels of engagement and low burnout would have higher levels of happiness and satisfaction with life than profiles with low levels of engagement and high burnout. When we analyzed the means of happiness and satisfaction with life (see Table 6 and Table 7) in the different profiles, we observed that, in both samples, the “Engaged” profile presented with levels of happiness and satisfaction with life higher than the “Burned-out” profile. Thus, H2 was supported.

With respect to Hypothesis 3, which presupposed that the profiles with high levels of engagement and high levels of burnout would have higher levels of happiness and satisfaction with life than profiles with low levels of engagement and low levels of burnout, the hypothesis cannot be analyzed since no such profiles were found. However, it was observed that the profiles that maintained similar levels of burnout but different levels of engagement (profiles 1 and 3 in SAS, and profiles 2 and 4 in TRAS) had higher levels of satisfaction with life and happiness when engagement was higher. This result shows that engagement can have a mitigating effect on the negative effects of burnout.

By analyzing the ANCOVA tests, we found that none of our control variables (sex, age and tenure) are significant for this study in relation to happiness and satisfaction with life. Regarding happiness in the SAS, these were the results: sex: *F* = 1.29 (n.s.); age: *F* = 0.11 (n.s.); tenure: *F* = 0.06 (n.s.); *p* < 0.005. For satisfaction with life in SAS, the following were the results: sex: *F* = 6.4 (n.s.); age: *F* = 0.05 (n.s.); tenure: *F* = 0.06 (n.s.); *p* < 0.005. Concerning happiness in TRAS, we found: sex: *F* = 1.25 (n.s.); age: *F* = 1.12 (n.s.); tenure: *F* = 0.07 (n.s.); *p* < 0.005. For satisfaction with life in TRAS, we found: sex: *F* = 10.26 (n.s.); age: *F* = 0.19 (n.s.); tenure: *F* = 4.4 (n.s.); *p* < 0.005. A summary of the hypotheses and main findings is presented in Table 8.

## 4. Discussion

One of the most significant contributions of this study is that two distinct populations (TRAS and SAS), who perform their work in the same context, were both grouped into four profiles, i.e., despite being different professions, well-being distribution was similar. In the four profiles in both samples, the same profile was dominant; the profile we called “Moderate well-being”, with high levels of engagement and moderate burnout. The literature considers that stress is very present in the work context [78]; in our study, the most populous profile consisted of workers with moderate levels of burnout and high engagement. It can, therefore, be concluded that, in this work context, the majority of workers experience well-being in their work. These results agree with those found by Chambel y Peiró [79] in a sample of human services workers. However, in the present study, it also found that some workers had low levels of well-being and even discomfort. Therefore, the idea of intervening in the work context to improve well-being at work is reinforced. In addition, co-occurrence in the same situation of burnout and engagement are evidenced, both being defined as components of well-being at work [13], as has been shown in previous studies [10].

It is also interesting to note that, by relating the variables that make up well-being at work with happiness and satisfaction with life, those workers who are engaged in their work and with low levels of burnout tend to be happier and satisfied with their lives outside the work context. Previous studies showed the independent relationship between burnout and life satisfaction [56,57] and happiness [54,55], and the relationship between engagement and satisfaction with life [60,61] and happiness [58,59]. However, the contribution of this study is that burnout and engagement, considered jointly, can explain the “context-free well-being” of university workers.

In addition, we can conclude that engagement sometimes acts as a mitigating factor. In the profiles with burnout, it is engagement that marked an improvement in these profiles, i.e., it reduced the negative effect of burnout. Based on this idea, it follows that it is essential to understand well-being at work from an integrative point of view, which includes a positive dimension (engagement) and a negative one (burnout) [80,81]. By simply bringing together the two variables, the relationship they have with well-being at work can be understood [13,82]. Finally, this study helps us to keep in mind that, when a decision is taken to make an intervention related to labor welfare, work should not be limited to burnout, because we have observed that at least as much attention should be given to engagement.

To conclude, some limitations contained in this study should be noted. First, data were collected by self-reporting measures, which may cause problems of bias. The nature of the factors of psychosocial risks make the establishment of procedures for objective and reliable measurement more difficult because, unlike other environmental and physical hazards or chemicals, psychosocial risks are rarely measured by some external characteristic of the individual, as pointed out by Benavides et al. [83]. Another limitation is that we have not analyzed longitudinal relationships between variables. As it is a cross-sectional study, we cannot establish causal relationships between variables. Finally, a suggestion for the future would be that transcultural and intercultural research on chronic work stress is needed. According to Gil-Monte [84] (p. 36), “future research should be directed to methodological changes that incorporate multilevel studies, longitudinal and research designs as well as an applied perspective to the results”. In short, in future research, it would be desirable to carry out longitudinal studies to address the temporal evolution of both organizational and personal and emotional variables, which would facilitate the identification of risk factors and protective factors concerning vulnerability to stress and well-being. Finally, this research should also be carried out in more professional groups and accompanied with action plans to achieve performance results in companies.

## 5. Conclusions

To summarize the study, it should be noted that most of the employees referred to in this research experienced well-being in their jobs. Furthermore, the co-occurrence of burnout and engagement occurred in the same situation and in the same context of work. Finally, with regard to happiness and satisfaction with life, engagement sometimes serves as a mitigating factor, i.e., reducing the negative effect of burnout levels.

In short, although burnout and engagement co-occur in the same context, this does not prevent the employee from developing well-being at work.

Moreover, given that engagement, with respect to happiness and life satisfaction, mitigates the negative effect of burnout, we believe that companies should pay special attention to this point, given that if they manage to keep their employees engaged in their jobs, the tendency will be for them to suffer less burnout. Likewise, if the employee strives to be more committed to their job, they will suffer less burnout.

Finally, having shown that there are different profiles, this suggests that companies should, as far as possible, give personalised attention to their employees in order to improve their levels of well-being at work.

## Figures and Tables

**Table 1:** Demographic data.

		SAS (*n* = 266)	TRAS (*n* = 319)
Gender			
	Men	116	193
	Female	150	126
Age			
	<30	13	30
	31–40	65	99
	41–50	115	100
	51–60	65	62
	>60	8	18
Job experience			
	<3	23	42
	3–5	23	54
	5–15	88	88
	15–20	49	40
	>20	83	95

**Table 2:** Means, standard deviations and correlations between variables.

	SAS (*n* = 266)		TRAS (*n* = 319)		1.	2.	3.	4.	5.	6.	7.	8.	9.	10.	11.
	Mean	*SD*	Mean	*SD*	*r* for SAS (Below the Diagonal) and For TRAS (Above the Diagonal)										
Gender	0.44	0.50	0.61	0.49		0.15 **	0.18 **	−0.15 **	0.03	−0.02	0.04	0.12 *	0.04	0.11	0.21 **
2. Age	2.96	0.90	2.75	1.08	0.18 **		0.75 **	−0.06	−0.04	−0.03	−0.01	−0.01	0.02	0.08	0.13 *
3. Job experience	3.55	1.25	3.29	1.39	0.14 *	0.65 **		−0.08	−0.08	−0.05	−0.01	−0.01	−0.00	0.08	0.16 **
4. Exhaustion	2.24	1.06	2.20	1.07	−0.03	0.00	−0.70		0.56 **	−0.32 **	−0.48 **	−0.45 **	−0.13 *	−0.29 **	−0.46 **
5. Cynicism	1.35	0.96	1.17	0.98	0.04	0.03	−0.05	0.71 **		−0.33 **	−0.20 **	−0.24 **	−0.11	−0.10	−0.24 **
6. Prof. Efficacy	3.82	0.72	4.21	0.79	0.02	0.06	0.08	−0.41 **	−0.38 **		0.57 **	0.62 **	0.44 **	0.38 **	0.44 **
7. Vigor	3.96	1.09	4.38	1.05	0.01	0.06	0.08	−0.60 **	−0.40 **	0.53 **		0.86 **	0.61 **	0.46 **	0.58 **
8. Dedication	3.91	1.20	4.62	0.97	0.03	0.05	0.02	−0.61 **	−0.42 **	0.54 **	0.85 **		0.66 **	0.50 **	0.62 **
9. Absorption	3.93	1.05	4.47	0.94	0.00	0.13 *	0.07	−0.46 **	−0.35 **	0.51 **	0.79 **	0.82 **		0.26 **	0.37 **
10. Happiness	4.64	1.14	4.94	1.05	0.07	0.00	0.02	−0.37 **	−0.23 **	0.21 **	0.43 **	0.38 **	0.22 **		0.63 **
11. Satisfaction with life	4.67	1.13	5.15	0.94	0.15 *	0.06	0.06	−0.49 **	−0.40 **	0.27 **	0.51 **	0.51 **	0.33 **	0.73 **	

* *p* < 0.05; ** *p* < 0.01.

**Table 3:** SABIC (Sample-Size Adjusted BIC) (SAS/TRAS).

2 profiles	4068.770/4798.940
3 profiles	3814.910/4536.157
4 profiles	3690.740/4456.501

**Table 4:** Number of individuals for each model of profiles (SAS/TRAS).

	1	2	3	4
2 profiles	160/236	106/83		
3 profiles	35/42	97/166	134/111	
4 profiles	83/40	34/93	119/48	30/138

**Table 5:** Average latent class probabilities for most likely latent class membership (row) by latent class (column) (SAS/TRAS).

	1	2	3	4
1	**0.963/0.921**	0.000/0.074	0.000/0.006	0.037/0.000
2	0.000/0.038	**0.941/0.899**	0.006/0.000	0.054/0.063
3	0.000/0.031	0.026/0.000	**0.974/0.969**	0.000/0.000
4	0.039/0.000	0.036/0.085	0.000/0.000	**0.926/0.915**

**Table 6:** Means associated with four-profiles model (SAS).

Profile	Happiness	Satisfaction with Life
1. Balanced well-being	4.6	4.57
2. Lack of well-being at work	3.46	3.26
3. Moderate well-being	4.94	4.96
4. Well-being at work	4.86	5.37
Post hoc comparisons ^a^	3 > 4 > 1 > 2	4 > 3 > 1 > 2

^a^ The post hoc comparisons indicated that the mean profile differs significantly at *p* < 0.05.

**Table 7:** Means associated with four-profiles model (TRAS).

Profile	Happiness	Satisfaction with Life
1. Lack of well-being at work	3.94	3.94
2. Balanced well-being	4.63	4.81
3. Well-being at work	5.62	6.03
4. Moderate well-being	5.20	5.41
Post hoc comparisons ^a^	3 > 4 > 2 > 1	3 > 4 > 2 > 1

^a^ The post hoc comparisons indicated that the mean profile differs significantly at *p* < 0.05.

**Table 8:** Summary of hypotheses and findings.

Hypothesis 1	Four Profiles	Findings
	High Engagement and Low Burnout	Supported
	High Burnout and Low Engagement	Supported
	Low Burnout and Low Engagement	Not Supported
	High Burnout and High Engagement	Not Supported
Hypothesis 2		
	High Engagement and Low Burnout -> More Happiness and Satisfaction with Life than Low Engagement and High Burnout	Supported
Hypothesis 3		
	High Engagement and High Burnout -> More Happiness and Satisfaction with Life than Low Engagement and Low Burnout	Not applicable, but Engagement mitigates Burnout
